# Field-applicable simultaneous multiplex LAMP assay for screening HBV and HCV co-infection in a single tube

**DOI:** 10.1186/s12879-024-09567-8

**Published:** 2024-08-09

**Authors:** Esra Agel, Kevser Hanne Altın

**Affiliations:** 1 Scientific and Technological Research Council of Türkiye, Sensor Technologies Research Group TUBITAK Marmara Research Center, Gebze, Kocaeli 41470 Türkiye; 2 Department of Medical Microbiology, Istanbul Medipol University, Beykoz, Istanbul 34815 Türkiye

**Keywords:** Multiplex LAMP, Isothermal amplification, Lateral flow dipstick, Rapid diagnostics, Point of care

## Abstract

**Background:**

Globally, around 7 to 20 million people are believed to be suffering from coinfection with both hepatitis B virus (HBV) and hepatitis C virus (HCV). The loop-mediated isothermal amplification (LAMP) approach, introduced by Notomi and colleagues, has undergone substantial advancements as an effective molecular tool that enables the simultaneous analysis of multiple samples in a single tube.

**Methods:**

The present study examined the simultaneous detection of HBV and HCV in a single tube using melt curve analysis multiplex LAMP (mLAMP), which is based on the identification of unique melting peak temperatures. Selected regions for primer design including the S gene of HBV and the UTR gene of HCV. Primer optimization is initially performed through individual HBV and HCV LAMP analysis. Following the optimization process, the mLAMP assay was evaluated by optimizing the multiplex reaction mixture, determining the reaction time, and analyzing the limit of detection (LOD). The results are also analyzed using lateral flow dipsticks (LFD), which enable the visual detection of HBV and HCV by adding 20 pmol FITC-labeled LF primers into the reaction mixture prior the mLAMP.

**Results:**

The LOD for the mLAMP assay was determined as 10 copies/µl, and no cross-reactivity with other microorganisms was detected. The detection results obtained from patient plasma were also visually demonstrated using LFD, and displayed significant concordance with those obtained from Real-Time Polymerase Chain Assay. The mLAMP assay revealed a diagnostic sensitivity of 95% for detecting the HBV, and LOD is 90% for HCV. The overall diagnostic sensitivity of the mLAMP assay for both viruses was 85%. The assay confirmed a specificity of 100%.

**Conclusion:**

The mLAMP assay displays significant promise for analyzing coinfected samples by simultaneously detecting the dual targets HBV and HCV within a set temperature of 62 °C, all within a time frame of 1 h. Additionally, when paired with disposable LFD, the mLAMP assay enables rapid visual detection of assay results in a matter of minutes. The result contributes to the mLAMP assay being highly suitable for coinfection screening, particularly in field conditions.

## Background

Viral hepatitis is a substantial challenge for developed and developing countries worldwide. According to data from the World Health Organization (WHO), 356 million individuals worldwide are infected with the hepatitis B virus (HBV), and 70 million individuals are infected with the hepatitis C virus (HCV) [1]. HCV infection plays a crucial role in coinfection since it is associated with the latent HBV infection observed in 50% of chronic HCV patients. Patients experiencing coinfection face an elevated risk of developing progressive liver disease, cirrhosis, and hepatocellular carcinoma compared to those with monoinfection [2]. Consequently, screening tests should simultaneously provide results for more than one pathogen using a single sample. The introduction of nucleic acid amplification technology has revolutionized disease diagnosis, pioneering loop-mediated isothermal amplification (LAMP), an assay that bypasses thermal cycling to amplify its target [3]. Although the use of the LAMP assay for diagnosing various pathogens has been explored, few studies have directly focused on diagnosing multiple pathogens in a single tube. Like with the real-time PCR (RT‒PCR) technique, multiplex diagnosis is performed using target-specifically designed probes in LAMP analysis [4]. The design of primers for the multiplex LAMP assay (mLAMP) can be complex, requiring specificity for the respective targets and preventing cross-reactivity with other sequences. Replication can also be initiated from both DNA and RNA. The integration of reverse transcription (RT) with LAMP has been applied to enable nucleic acid amplification in the detection of the RNA genome with DNA, resulting in the development of reverse transcription LAMP [5]. The primary enzyme involved in this process is reverse transcriptase (RT), which efficiently amplifies RNA and DNA in a single tube, removing the necessity for an additional process of synthesizing copy DNA [6].

The main constraint of the LAMP assay lies in the proper design of the primer. Therefore, multiplexing approaches for LAMP are advanced than conventional PCR [7]. In many studies on mLAMP assay, probe-based assays, which are laborious and complex, have been attempted [8, 9]. In our study, mLAMP assay was based on simultaneous melting curve analysis. Various methods can be employed for detecting LAMP products, including gel electrophoresis, turbidity detection caused by reaction by products, colorimetric changes through adjusting the reaction pH, and the utilization of lateral flow strips. The lateral flow dipstick (LFD) operates on the basis of the antigen‒antibody reaction principle. However, recently, the LFD system based on DNA hybridization has been utilized to visualize amplification products [10]. Therefore, in our study, we also aimed to perform the mLAMP with a simple heater in the same tube suitable for field use and to display the results using LFD.

## Methods

### Design of the HBV and HCV LAMP primers

The best characterised member of the Hepacivirus genus is hepatitis C virus (HCV), classified as a member of the species Hepacivirus hominis. All HCV genotypes are included in this Hepacivirus hominis species. When selecting the conserved region, Hepacivirus hominis was chosen to ensure ease of screening among all genotypes and subtypes [11]. The complete genome sequence of the target viruses was obtained from the GenBank National Center for Biotechnology Information (NCBI, Maryland, USA). The HBV genome with the ID MZ093431.1 was selected through the use of a nucleotide search tab on the NCBI website. The FASTA format information of the HBsAg (S protein) region was obtained. The conserved 5’UTR, common to all HCV genotypes and subtypes in the Hepacivirus hominis genus, was selected for the design of the LAMP primer for the Hepatitis C virus. Similarly, the HCV genome FASTA format of the region spanning nucleotides 35 to 207, linked to the gene ID OQ873161.1, was downloaded from the NCBI website.

For the design of the LAMP primers, PrimerExplorer V5 software (Fujitsu Limited, Tokyo, Japan) was used. All the designed primers were bioinformatically checked against the NCBI database to prevent cross-reactions and false positives. Quality control was performed with Integrated DNA Technologies (IDT) OligoAnalyzer online software (Coralville, IA, USA). From the available primer sets, the one displaying a Delta G (ΔG) value in close proximity to the positive value was selected. The secondary structure of the LAMP primers was then verified by comparing the reference ΔG values given by the IDT program. The LAMP primers used were manufactured by Oligomer Biyoteknoloji (Ankara, Türkiye) and were lyophilized. The primer stocks were then diluted with 1X TE buffer according to the prescribed conditions and preserved at a temperature of -20 °C. The primers used in the study are shown in Table 1.

**Table 1:** The HBV and HCV primer sequences used in the study

Primer setting	Primer sequence 5’ to 3’	Length(bp)	G/C%	
*HbS-F3	TCCAGGATCATCAACCACCA	20	50	
HbS-B3	CGAACCACTGAACAAATGGA	20	45	
HbS-FIP	GGTGCAGTTGCATCCGAAGGTCAGAACCTGCGCACTCC	38	60.5	
HbS-BIP	ATTCCCATCCCATCGTCCTGGGCAAGAGAAACGGGCTGAGG	41	58.5	
HbS-LF	GGATACATAGAGGTTCCTTGAGCA	24	45.8	
HbS-LB	CTTTCGGAAAATCCTATGGGAGT	23	43.5	
HCV-F3	TCACTCCCCTGTGAGGAAC	19	58	
HCV-B3	GGGTTGCTCCAAGAAAGGAC	20	55	
HCV-FIP	GAGGCTGCACGACACTCGTTTTTCTGTCTTCACGCGGAAAG	41	53.7	
HCV-BIP	CCTCCCGGGAGAGCCATAGTTTTGGTCACCCCAGCGATTCC	41	61	
HCV-LF	GCCATGGCTAGACGCT	16 62.5		
HCV-LB	AGCGTCTAGCCATGGC	16	62.5	

*HbS: Hepatitis B surface protein

### HBV and HCV LAMP assay optimization

Before the optimization of multiplex LAMP, individual optimizations were conducted for the LAMP protocols for HBV [12] and HCV. The optimization process involved adjusting several parameters. First, the ratio of the inner primers to the outer primers was varied, ranging from 1:4 to 2:1. Amplification temperatures were determined with a T100 Thermal Cycler (Bio-Rad, USA) by setting up a temperature ranging from 60 °C to 70 °C. Once the reaction temperature was established, the results were examined to determine the optimal duration for the procedure, involving time intervals of 30, 35, 45, 50, and 60 min.

All the reactions were conducted in triplicate to ensure consistency, and the amplified products were visualized by 2.5% agarose gel electrophoresis. The HBV and HCV primers used were optimized in terms of primer quantity, nucleic acid concentration, time, and temperature. The optimal reaction mixtures for a total volume of 25 µL are provided in Table 2.

**Table 2:** Composition of reaction mixtures for individual HBV and HCV LAMP assay

Reaction mix	HBV (µl)	HCV (µl)
*WarmStart 2x master mix	12.5	12.5
Primer mix	2.5	4.5
*LAMP fluorescent dye	0.5	0.5
PCR grade water	8.5	4.5
DNA/RNA	1	3
Total volume	25	

*Purchased from New England Biolabs (Ipswich, USA)

### Multiplex LAMP reaction conditions and optimization

The mLAMP assay has been improved by applying separately optimized procedures for the HBV and HCV LAMP assays. The analysis of the mLAMP results involved the use of fluorescent dye, which can bind to double-stranded DNA during each amplification cycle. Subsequently, a melting temperature (Tm) analysis was carried out to observe the separation of the dye from the DNA at particular temperatures, resulting in the emission of radiation. A CFX96 Touch Real-Time PCR system (Bio-Rad, USA) was used to detect and analyze the released radiation. For multiplex LAMP, temperature gradient and optimization studies for the reaction time were also conducted. To achieve this goal, three separate protocols were developed, each optimized to target the right diagnostic procedure. The protocol determination analyses were performed on the same day using identical samples, after which the findings were compared data not shown. The multiplex primer mixture was obtained by 2.5 µL of the HBV and 1.25 µL of the HCV LAMP primer sets. The concentrations of the primer sets for both viruses used were as follows: 4 µM F3, 4 µM B3, 16 µM FIP, 16 µM BIP, 8 µM LF and 8 µM LB. The assay that has been optimized and confirmed is displayed in Table 3.

**Table 3:** Composition of the mLAMP reaction mixture (single tube for HBV and HCV)

mLAMP Reaction mix		µL
*WarmStart 2X master mix		12.5
Multiplex primer mix	HBV	2.5
	HCV	1.25
LAMP fluorescent dye		0.5
PCR grade water		5.25
†Nucleic acid		3
Total volume		25

* Purchased from New England Biolabs (Ipswich, USA)

†Nucleic acid: Contains HBV DNA and HCV RNA

### LAMP product visualization with lateral flow dipstick

**Fig. 1:**
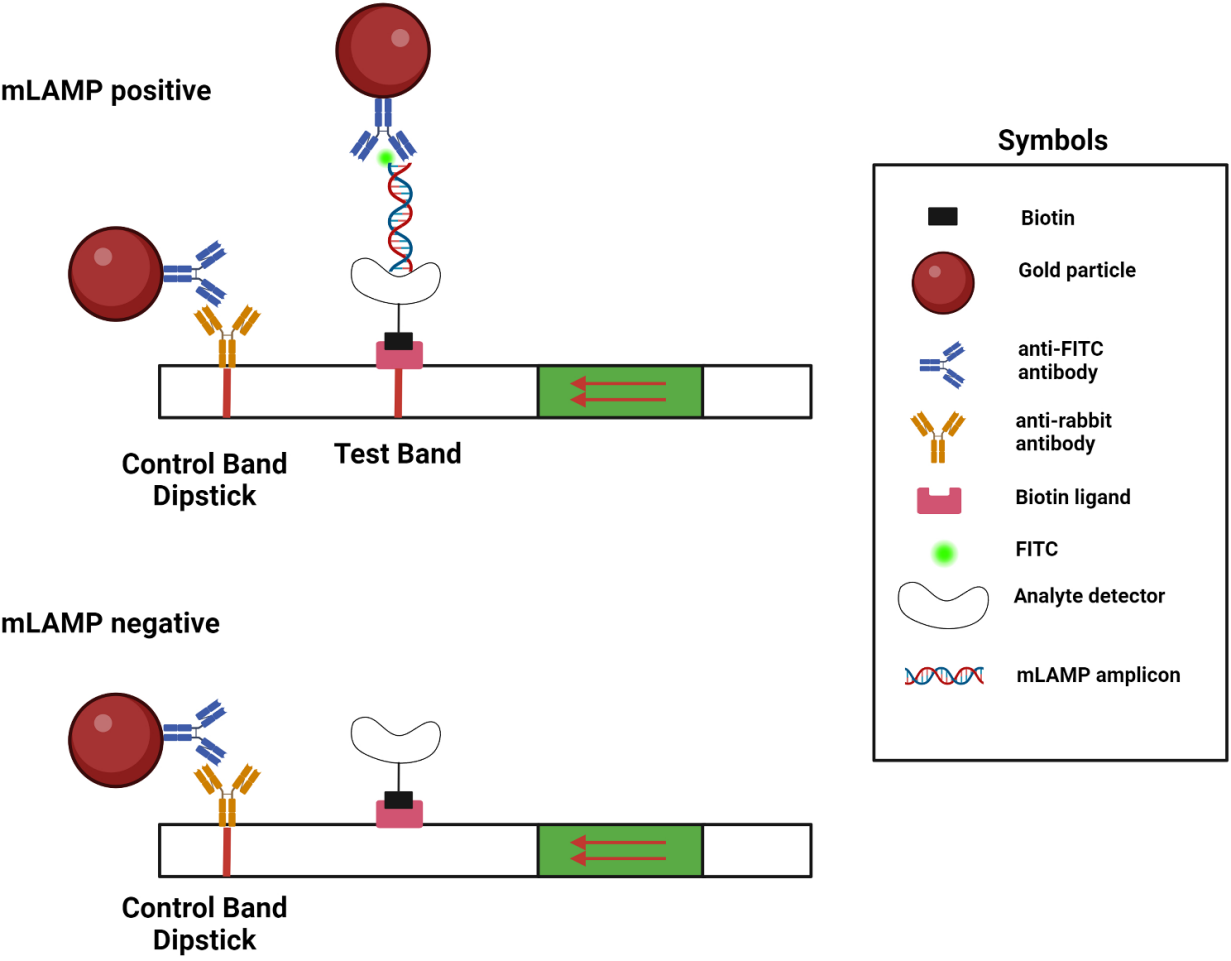
Test mechanism for displaying the LAMP analysis result with LFD

LAMP products were visualized by using the Milenia HybriDetect LFD. For the LFD analysis, FITC-labeled LF primers were used: 5’-end GGATACATAGAGGTTCCTTGAGCA-FITC for HBV and 5’-end GCCATGGCTAGACGCT-FITC for HCV. Those primers were manufactured by Oligomer Biotechnology (Ankara, Türkiye). LFD analysis was conducted in accordance with the manufacturer’s instructions (Milenia Biotec, Gießen, Germany). 20 pmole FITC-labeled HBV-HCV primers were added to mLAMP reaction mix. After amplification, 10µL the mLAMP product was transferred into a new sterilized Eppendorf tube that already contained 100 µL of HybriDetect assay buffer. The mixture was then incubated in an upright position for 5 min at room temperature, as described in the manufacturer’s protocol. Following the incubation process, the final product was tested by immersion with a test strip, and the outcome that resulted was observed immediately. Since the HBV-LF and HCV-LF primers used in the mLAMP assay labelled with FITC, the mLAMP-LFD limit of detection analysis result is the equivalent to that of the mLAMP assay with melting peak. Consequently, the limit of detection for mLAMP-LFD is 10 copies/µL. The test mechanism for displaying LAMP results with LFD is shown in Fig. 1.

### The sensitivity and specificity analysis of clinical samples

Sensitivity and specificity analysis of the mLAMP assay results with clinical samples were calculated according to the formulas below;

$$Sensitivity\left(\%\right)=\frac{True\;positive}{True\;positive+False\;negative}x100$$

$$Specificity\left(\%\right)=\frac{True\;False}{True\;False+False\;positive }x100$$

### Validation and application

HBV and HCV seropositive plasma samples were obtained from Istanbul Medipol University Genetic Diseases Evaluation Center (MEDIGEN, Türkiye) under cold chain conditions. During the period of sample collection, no coinfected patients were found. Thus, to validate these results with clinical samples, simulant co-infected samples were generated by combining HBV and HCV plasma with known cycle threshold (Ct) values (ranged from 21 to 32) in a one-to-one ratio and those samples were isolated using the DNeasy Blood and Tissue Kit (Qiagen, Germany). HBV-HCV viral loads were confirmed by using HBV and HCV RTA Real-Time PCR kits (RTA Laboratories, Türkiye) separately.

## Results

### HBV and HCV LAMP assay optimization

The HBV LAMP assay was optimized at a temperature of 62 °C for a duration of 45 min [12], while the optimization of the HCV LAMP was completed at 63.8 °C in 60 min. According to the melting peak analysis results, the peak melting temperature of HBV DNA was 85.00 ± 0.05 °C, while it was found to be 89.50 ± 0.05 °C for HCV RNA. The HBV LAMP assay was capable of detecting as few as 10 copies/µL in clinical plasma samples, with a sensitivity of 92.20%. Similarly, the HCV LAMP assay showed a limit of detection 10 copies/µL and a sensitivity of 91.6%.

### The mLAMP assay optimization results

**Fig. 2:**
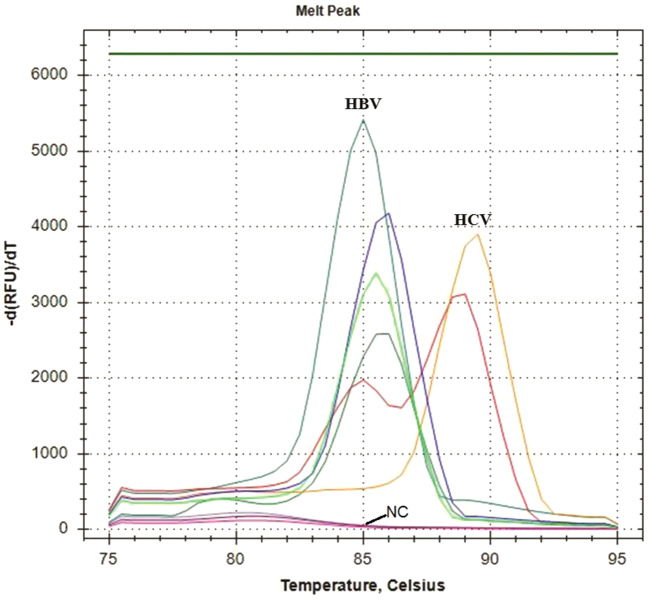
The mLAMP assay gradient analysis result *Peak color at specific temperature; Pink: NC. Purple: 70 °C. Lilac: 69.5 °C. Green: 68.4 °C. Light green: 66.4 °C. Orange: 64 °C. Red: 62 °C. Turquoise blue: 60.7 °C. Blue: 60 °C

**Fig. 3:**
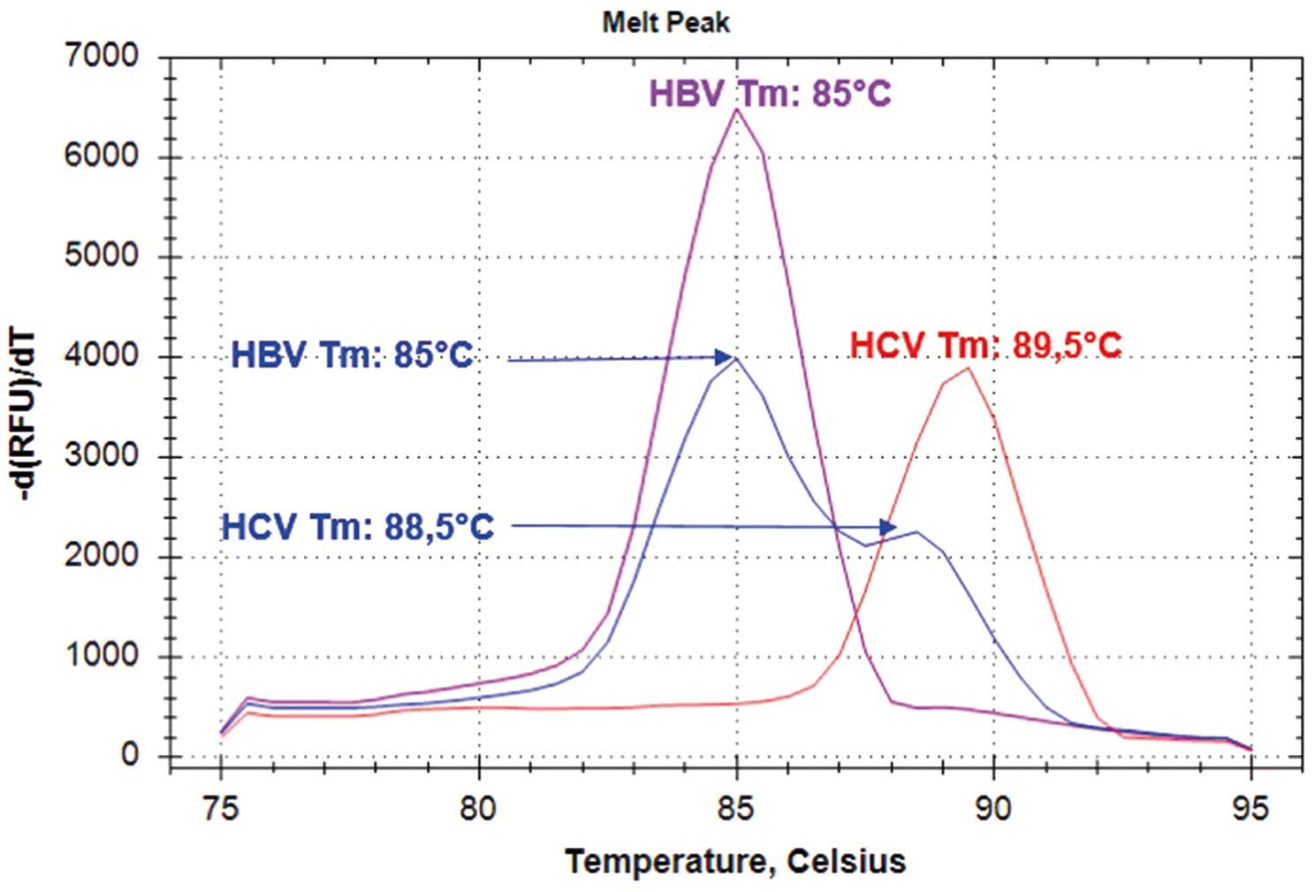
The graph displays the melting curve analysis of HBV-HCV individually along with the multiplex analysis. * The pink and red melt peak graphs correspond to the two different LAMP assays, whereas the blue melt peak graphs provide the Tm values for mLAMP assay

A gradient analysis was carried out within the temperature range of 60–70 °C to determine the optimal reaction temperature for the simultaneous activity of HBV and HCV in a single tube (Fig. 2). The research revealed that the overall temperature for detecting of both viruses was 62 °C, and the optimal duration was 60 min. Analysis of the melting temperature peak for multiplex HBV-HCV LAMP demonstrated that the melting temperature peak for HBV was 85.00 ± 0.25 °C, but the melting temperature peak for HCV was 88.50 ± 0.20 °C (Fig. 3).

### Limit of detection of the mLAMP assay

**Fig. 4:**
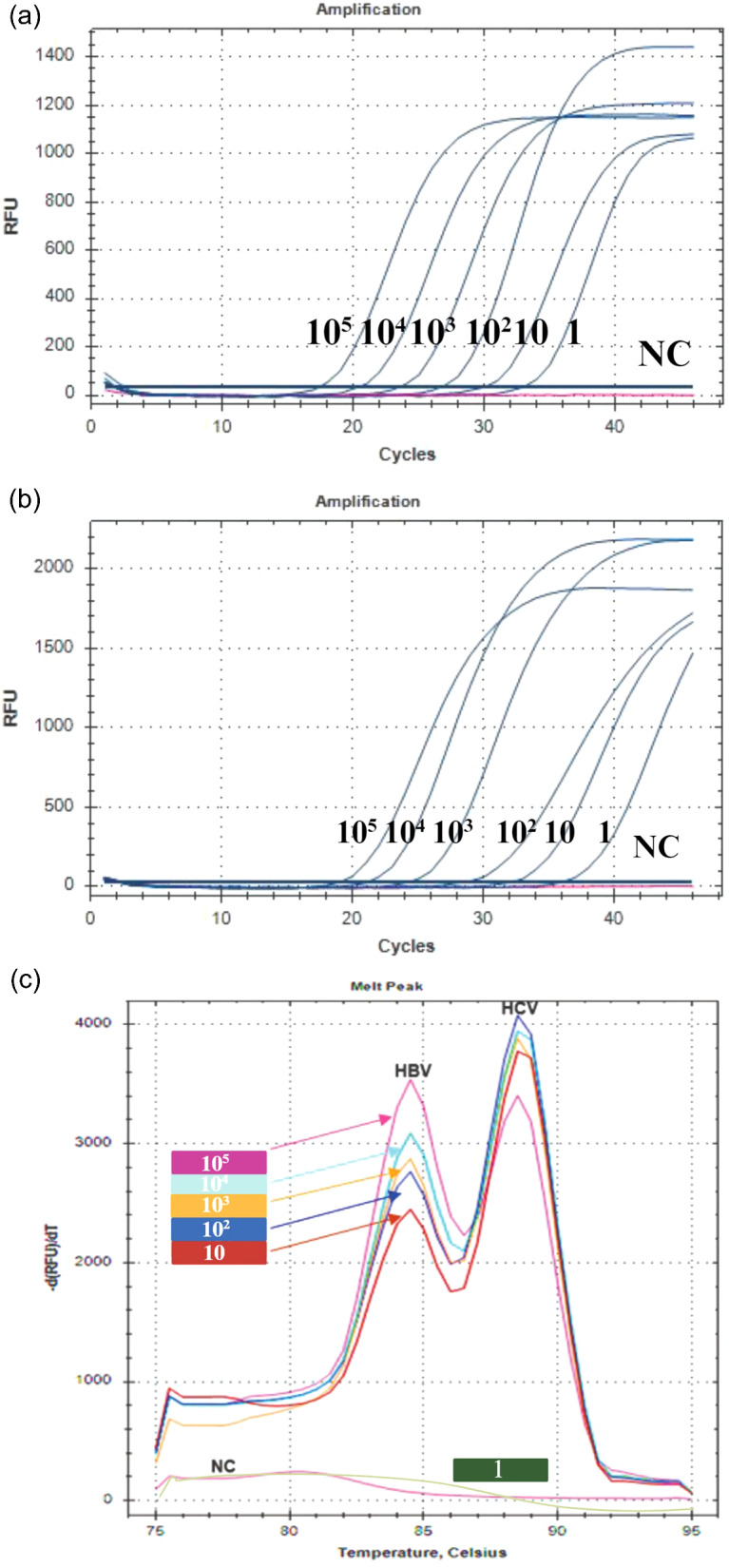
(**a**) HBV ATCC serial dilutions RT-PCR amplification graph. *10^5^ copies/µL, 10^4^ copies/µL, 10^3^ copies/µL, 10^2^ copies/µL, 10 copies/µL NC, Negative control. (**b**) HCV ATCC serial dilutions RT-PCR amplification graph. *10^5^ copies/µL, 10^4^ copies/µL, 10^3^ copies/µL, 10^2^ copies/µL, 10 copies/µL NC, Negative control. (**c**) The colors represent the melting peaks of serial dilutions of the ATCC HBV and ATCC HCV performed with the mLAMP assay. *10^5^ copies/µL, 10^4^ copies/µL, 10^3^ copies/µL, 10^2^ copies/µL, 10 copies/µL, 1 copies/µL NC, Negative control

The limit of detection study of the mLAMP assay was conducted using serially diluted samples of 10^6^ copies/µL ATCC HBV DNA (VR-3232SD) and 10^6^ copies/µL ATCC HCV RNA (VR-3233SD), as shown in Table 4, ranging from 10^5^ to 1 copies/µL. The LOD was determined by a parallel analysis of HBV and HCV ATCC reference sample serial (10^− 1^, 10^5^ copies/µL; 10^− 2^, 10^4^ copies/µL; 10^− 3^, 10^3^ copies/µL; 10^− 4^, 10^2^ copies/µL; 10^− 5^, 10 copies/µL) dilutions. The standard curve result of analysis of serial dilutions of HBV and HCV ATCC reference samples by RT PCR is shown in Fig. 4a and b. Significant changes in the melting peaks were observed for the HBV and HCV samples with a viral concentration of 10 copies/µL demonstrating a sensitivity level comparable to that of RT-PCR. Nevertheless, the samples with a concentration of 1 copies/µL for both viruses, along with the negative control, failed to display any observable melting peak (Fig. 4c). Consequently, the detection sensitivity of mLAMP was determined to be 10 copies/µL (genomic HBV DNA and HCV RNA). The detection rate was confirmed by three repetitions on different days.

**Table 4:** Ct values ​​of the ATCC reference nucleic acid dilution series obtained with the *commercial RT-PCR kit

**Serial dilutions of NA	Copies/µL	HBV qPCR Ct value	HCV RT‒PCR Ct value
10^− 1^	10^5^	17.38	18.82
10^− 2^	10^4^	20.36	21.04
10^− 3^	10^3^	23.48	24.01
10^− 4^	10^2^	26.68	28.03
10^− 5^	10	30.05	31.78
10^− 6^	1	33.01	35.73

*RT PCR kit purchased from RTA (RTA Laboratories, Türkiye)

**Consecutive dilutions of ATCC HBV DNA and ATCC HCV RNA reference samples

### Specificity of the mLAMP assay

**Fig. 5:**
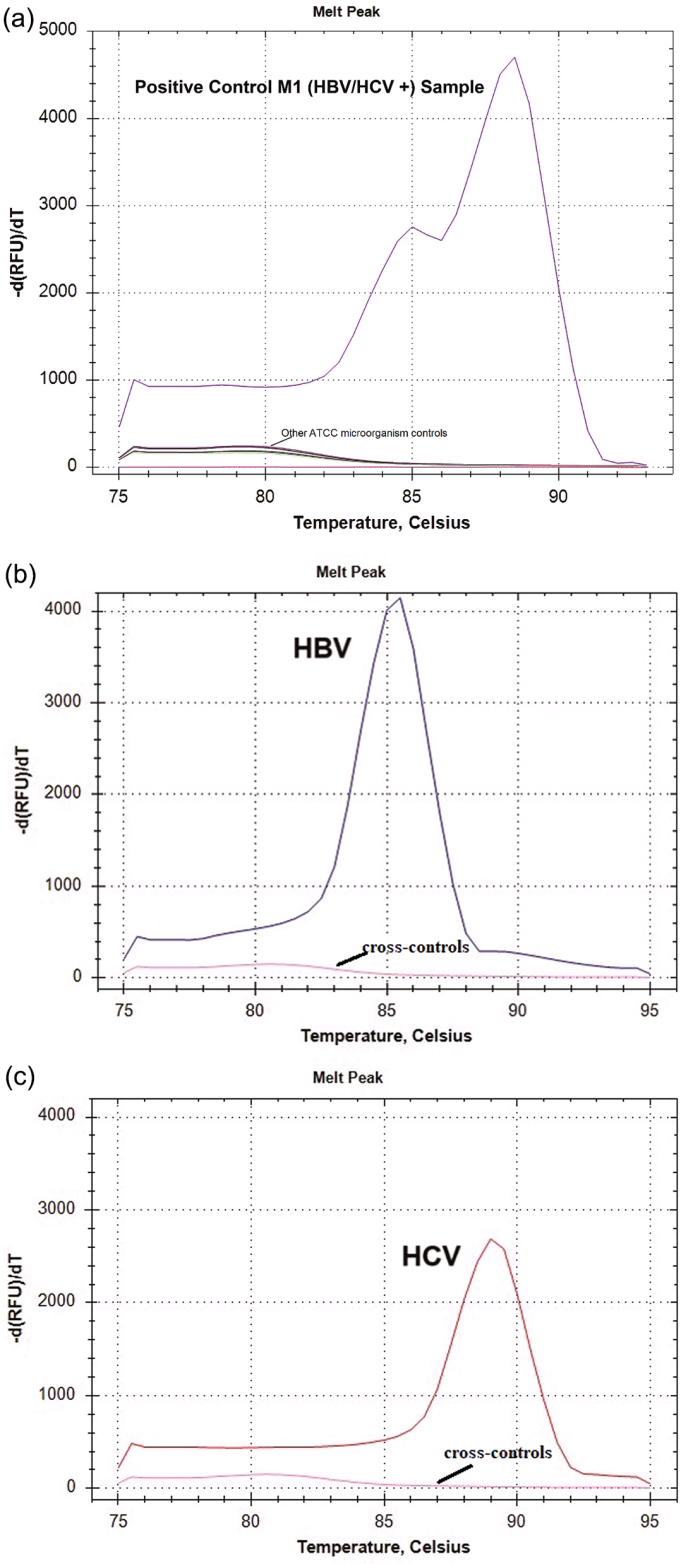
(**a**) The cross-reactivity of the mLAMP assay was verified by screening a) shows specificity of HBV and HCV primers against HIV-1, *E. coli*, *Salmonella enterica*, *Legionella pneumophila* and *Mycobacterium tuberculosis*. (**b**) Displays for HBV primary specificity analysis, analysis was performed with a co-infected sample and only a peak was observed at the HBV melting temperature value. (**c**) The same process was performed for the HCV primer control and only the HCV peak was observed

To demonstrate the accurate discrimination between positive and negative results for both virus primers, cross-reaction controls were conducted with various nucleic acid samples from HIV-1 (ATCC VR-3245SD), *E. coli* (ATCC 25,922), *Salmonella enterica* (ATCC 14,028), *Legionella pneumophila* (ATCC 33,152), and *Mycobacterium tuberculosis* (ATCC 27,294), shown in Fig. 5a. In addition, independent cross-controls were performed using HCV for HBV (VR-3232SD) and HBV for HCV (VR-3233SD), displayed in Fig. 5b and c.

### The mLAMP validation study with plasma samples

**Fig. 6:**
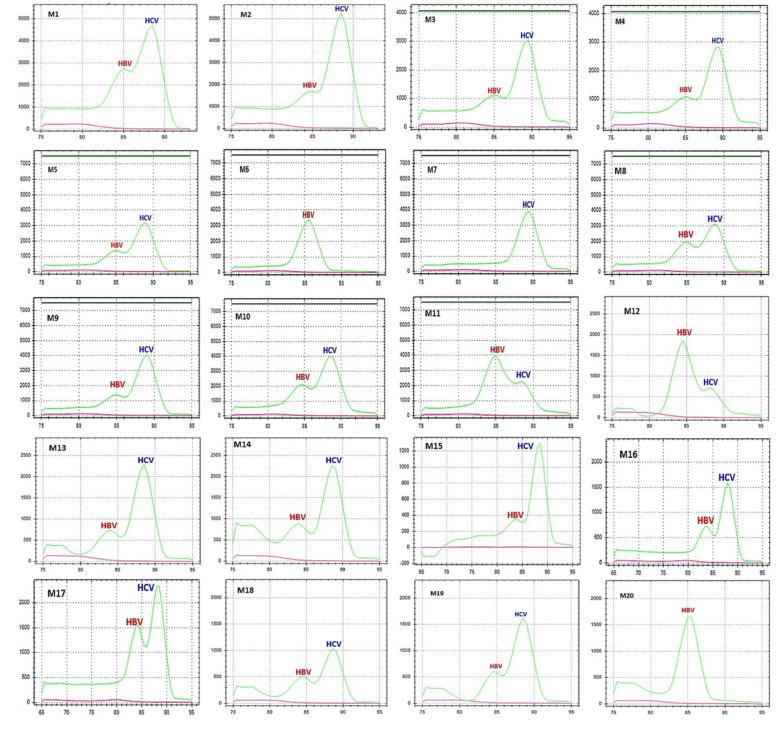
Validation of mLAMP assay on coinfection simulated samples

The optimized mLAMP procedure was applied for examining the 20 co-infected samples, as shown in Table 5. Figure 6 displays the graphical representation of the peak melt temperature generated from the study data. In this study involving samples M6, M7, and M20, shown that multiplex findings could not be achieved for HBV and HCV samples with Ct values over 31. However, multiplex results were obtained in the remaining 18 studies with Ct ≤ 30. The sensitivity of plasma samples were determined based on the positive, and negative findings obtained using the mLAMP assay. Out of 20 total positive samples, the mLAMP assay found 17 positive and 3 false negative. The total sensitivity of the assay was determined to be 85%. The failure of multiplex analysis in all three studies was thought to be possibly linked to patient plasma samples. Therefore, if the mLAMP HBV and HCV sensitivity studies were evaluated separately, the mLAMP assay displayed a sensitivity of 90% for diagnosing HCV samples and a sensitivity of 95% for identifying HBV samples.

**Table 5:** The ct values of HBV and HCV that constitute the mixed samples

HBV + HCV mix Sample* no	HBV Ct value	HCV Ct value
M1	28.11	22.9
M2	30.5	21.7
M3	30.38	26.7
M4	31	27.9
M5	30.18	25.7
M6	25.8	31.18
M7	33	22.9
M8	26.27	26.9
M9	31	27.9
M10	28.66	24.73
M11	18.17	28.17
M12	25.68	30.5
M13	31.31	29.2
M14	31.08	27.9
M15	31.8	30.43
M16	32.23	26.29
M17	27	25.62
M18	30.9	28.65
M19	31.88	30.86
M20	26.73	31.18

* The Ct values of these samples were established by RT-PCR analysis. The samples presented in this table were applied in the process of validating the study, and the results are displayed in Fig. 6

### Visualization of the mLAMP product by LFD

**Fig. 7:**
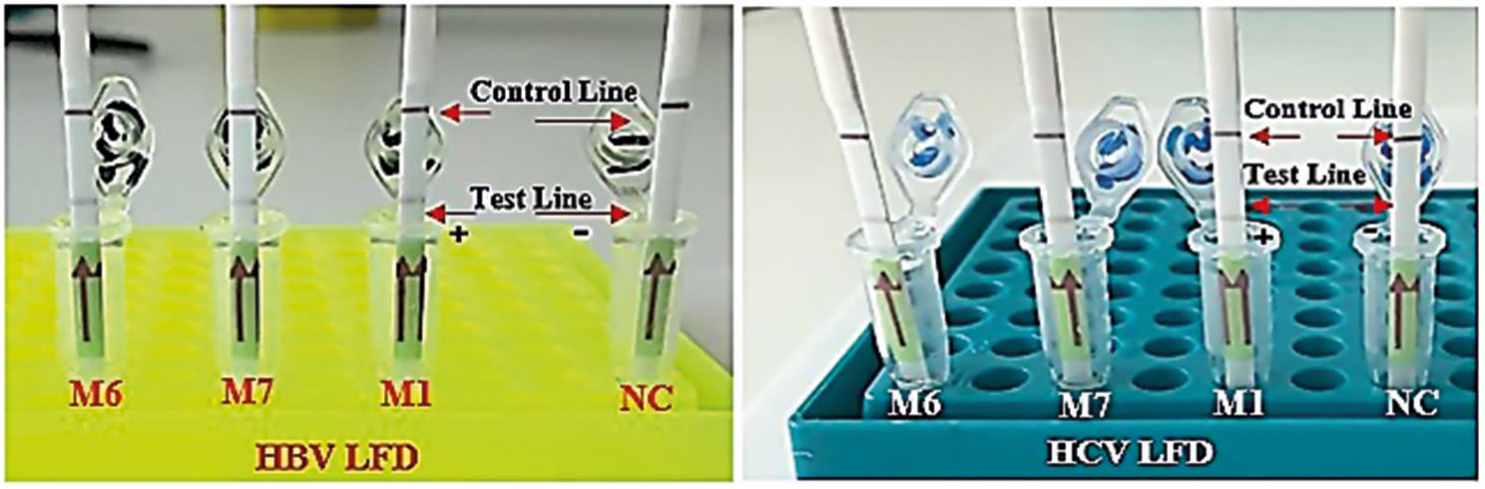
The visual outcomes of the mLAMP assay demonstrating a positive result on LFD

**Fig. 8:**
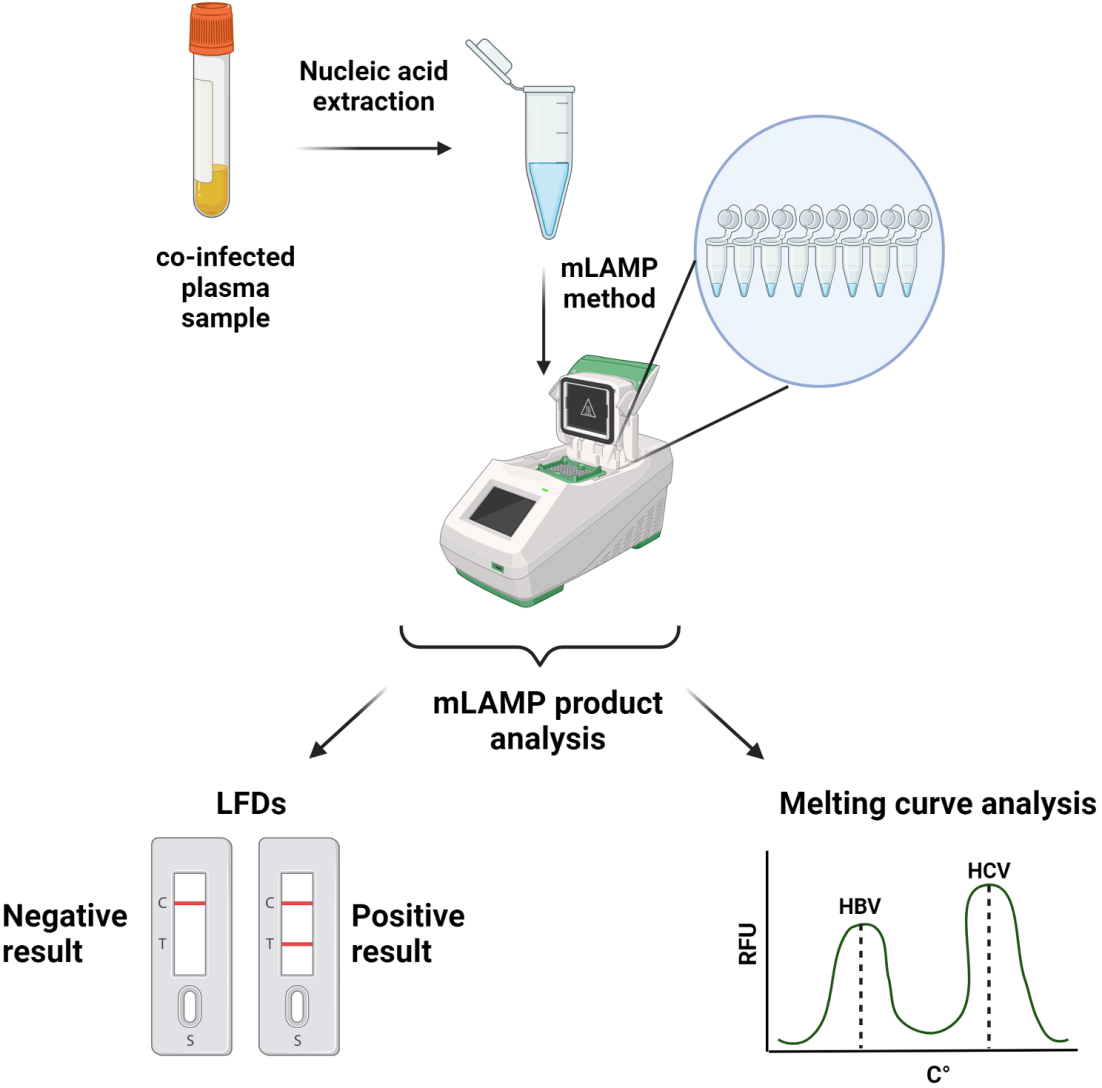
Summary flowchart of mLAMP analysis and display of results

Co-infected samples were observed within a few minutes on LFDs that were generated following mLAMP at 62 °C for 60 min. Figure 7 displays the LFD results for samples M1, M6, and M7, demonstrating the study’s findings. In the control analyses for HBV and HCV, test lines on the LFDs were obtained by adding FITC-labeled HBV LF and HCV LF primers separately into two different LAMP mixtures. Separate LFDs were used for each virus. The process of analyzing HBV and HCV with the LAMP method and displaying the results is summarized in Fig. 8.

**Table 6:** Details regarding numerous mLAMP studies performed between 2012 and 2021 for diagnosing various microorganisms

Target	Method	Visualization	*LOD	Reference no
*E. histolytica* T1,T2,T3	Probe Biotin, Texas red, Digoxigenin	NALFA strip	-	[23]
Dengue v. Chikungunya v.	Probe Biotin, Texas red, Digoxigenin	Naked eye, magnetic particle clumping	51.55 mg/µL	[24]
*T. pallidum* *Haemophilus ducreyi*	Mediator displacement probe	Cyto5 FAM	300 copies	[25]
*Salmolella* spp. *V. parahaemolyticus*	Tm values	Melt peak	1 ng	[22]
*Campylobacter* spp. *Salmonella* spp.	Probe Biotin, Digoxigenin	LFD	1 ng-100 pg	[26]
Cow milk Goat milk	Assimilating probe	Fluorescence	0.1 pg-1 pg	[27]
Influenza A(H1-H3) Influenza B	Tm values	Melt peak	1 genome equivalent (ge)	[28]
HBV HCV HIV *Toxoplasma pallidum*	Nease mediated pyrosequencing	Niced amplicon peak Pyrosequencing signals	-	[29]
West Nile v. Chikungunya v.	Quasr primer and complete mentary quencing probe FAM, HEX, Cyto5	SYTO dyes	-	[8]
Porcine Epidemic Diarrhea v. Porcine Circov type 2 v.	Probe Biotin, Digoxigenin	LFD	0.1 ng/µL 0.246 ng/µL	[9]
*V. vulnificus* *V. parahaemolyticus*	Probe Digoxigenin, Biotin	LFD	2.2 × 10^5^cfu/g	[30]

*LOD: limit of detection

## Discussion

The prevalence of HBV/HCV coinfection worldwide is approximately 7–20 million patients [13]. The risk of progressive liver disease, cirrhosis, and hepatocellular carcinoma is greater in coinfected patients than in patients with monoinfections [14]. Therefore, screening tests need to provide results for multiple pathogens simultaneously using a single sample. The LAMP technique is an excellent alternative to PCR; it is fast and user friendly and has high sensitivity and specificity without the need for skilled staff and expensive equipment and can be integrated with microstructured devices for point of care systems [15].

Despite the features stated above are common among isothermal amplification techniques, the LAMP assay distinguishes itself with several advantages over alternative methods. Other isothermal methods, including Nucleic Acid Sequence-based Amplification (NASBA), Recombinase Polymerase Amplification (RPA), Rolling Circle Amplification (RCA), Strand Helicase-dependent Amplification (HAD), and Multienzyme Isothermal Rapid Amplification (MIRA), require a combination of several enzymes to amplify the target region [16, 17].

However, following the LAMP assay, DNA amplification is only carried out using the Bst polymerase enzyme. An inappropriate amount of enzyme could also result in the assay turning into costly. The capability offers a benefit in cost-effective laboratory and emergency imaging. Furthermore, unlike other isothermal techniques, the LAMP approach provides high specificity in diagnosis due to the use of six unique primers that bind to eight separate targeted regions [3].

The use of random primers in the Multiple Displacement Amplification.

(MDA) assay limits the method’s sensitivity while elevating non-specific binding, leading to a method specificity that yields a success rate of 30% [17].

Due to the limited amplification of particular genome types, isothermal methods are unable to carry out numerous studies. In this regard, the NASBA assay only amplifies single-stranded DNA and RNA [18], while the RCA assay specifically amplifies circular single-stranded DNA and miRNA [17]. Nevertheless, the LAMP assay enables unrestricted amplification of DNA and RNA genomes using the reverse transcriptase enzyme [19].

The LAMP also effectively avoids false positive results because to its polymerase enzyme activity at high temperatures. However, with RPA and MIRA assays, enzymatic processes occurring at low temperatures might lead to inaccurate positive results [17]. The most significant advantage of the LAMP approach is the production of insoluble pyrophosphate in the solution during amplification [17, 20]. This enables a visual evaluation of the LAMP results.

Although the LAMP assay has been employed for the diagnosis of many pathogens, the number of studies specifically addressing the diagnosis of multiple pathogens of the same type is quite limited. Iseki et al. reported the use of the mLAMP in 2007 [21]. In mLAMP, a set of different primers is designed, each specific to one of the target regions. This set of primers included inner primers, outer primers, and loop primers for each target. Liu et al. reported a rapid LAMP and real-time monitoring procedure for the simultaneous detection of foodborne disease pathogens such as *Vibrio parahaemolyticus* and *Salmonella* spp. In a reaction tube, primer sets targeting these two pathogens were added, and after isothermal amplification for 60 min, the products were subjected to melting curve analysis [22]. It was possible to distinguish these two microorganisms through the difference in melting temperature (Tm) during the LAMP reaction. We have successfully combined mLAMP with melting curve analysis and LFD to provide a novel and efficient assay for detecting multiple infections of HBV and HCV in a single tube. However, like with the RT-PCR analysis, duplex diagnosis is also performed using target-specifically designed probes in LAMP assay [4]. In many studies on mLAMP, probe-based methods, which are laborious and complex, have been attempted [23]. Nevertheless, our assay facilitates result analysis using an ordinary intercalation dye, eliminating the necessity for expensive probes.

The distinct peaks in the mLAMP with melting curve graph for HBV and HCV enable rapid diagnosis without a requirement for further study. The relative fluorescence units (RFU) value presented on the graph enables the evaluation and interpretation of the hepatitis viral load in the patient. The mLAMP, which involves a quantitative diagnostic methodology, allows for the identification of individuals who are co-infected alongside those who have been diagnosed with HBV or HCV.

Furthermore, multiplex LAMP is defined as the assay in which the amplification results of multiple targets are displayed on the strip [23]. In cases where melting curve analysis cannot be conducted, the aim is to demonstrate that HBV and HCV result from the same tube using target-specific lateral flow devices (LFD). The mLAMP-LFD approach provides for the visual observation of positive and negative samples, enabling initial diagnosis. Yet, due to the limitation of LFD to provide a multiplex procedure, the detection of pathogen is restricted to a single line on LFD. Therefore, further diagnostic techniques such as molecular-based or serological methods are necessary to accurately diagnose HBV or HCV following the initial diagnosis. Despite this limitation, viral load in a patient blood could potentially be differentiated from that of healthy individuals by a preliminary diagnosis in rapid blood screening. Rest of studies aiming to simultaneously detect multiple targets using the LAMP assay are summarized in Table 6.

## Conclusions

The ability to identify multiple pathogens in a single analysis saves time and resources by reducing the need for laboratory manipulations. Multiplex LAMP provides the capability to analyze different pathogens simultaneously using a single assay. These advantages highlight the importance of multiplex LAMP, especially in situations where rapid and effective detection of multiple pathogens is crucial, such as in epidemiological studies or emergencies. Future research should include examining actual patients with co-infections to confirm the sensitivity of the test.

## Data Availability

All data generated or analyzed during this study are included in this published article.

## Abbreviations

ATCC: American Type Culture Collection
HBV: Hepatitis B virus
HBsAg: Hepatitis B Surface Antigen
HCV: Hepatitis C virus
Ct: Cycle Threshold
BIP: Backward Inner Primer
B3: Backward Outer
F3: Forward Outer
FIP: Forward Inner Primer
LF: Loop Forward
LB: Loop Backward
FASTA: Fast-All
HAD: Helicase-dependent Amplification
IDT: Integrated DNA Technologies
LAMP: Loop Mediated Isothermal Amplification
LOD: Limit of Detection
LFD: Lateral Flow Dipstick
mLAMP: Multiplex Loop Mediated Isothermal Amplification
MIRA: Multienzyme Isothermal Rapid Amplification
MDA: Multiple Displacement Amplification
NC: Negative Control
NCBI: National Center for Biotechnology Information
NASBA: Nucleic Acid Sequence-based Amplification
POCT: Point of Care Testing
RPA: Recombinase Polymerase Amplification
RCA: Rolling Circle Amplification
RT: PCR-Real Time Polymerase Chain Reaction
RT: LAMP-Reverse Transcription LAMP
RFU: Relative fluorescence units
SDA: Strand Displacement Amplification
TE: Tris EDTA
5’UTR: 5’ Untranslated Region
USA: United States of America
WHO: World Health Organization
